# A Critical Combination of Esophageal Rupture and Upside-down Stomach: A Case Report

**DOI:** 10.5811/cpcem.20907

**Published:** 2024-04-17

**Authors:** Kay Nicole Tipton, Daniel Schroder

**Affiliations:** * UNC Health Southeastern, Department of Emergency Medicine, Lumberton, North Carolina; † Campbell University, School of Medicine, Emergency Medicine Residency, Lumberton, North Carolina

**Keywords:** esophageal rupture, Boerhaave syndrome, upside-down stomach, hiatal hernia, case report

## Abstract

**Introduction:**

Spontaneous esophageal rupture, or Boerhaave syndrome, and upside-down stomach are rare pathologies associated with grave sequelae. Boerhaave syndrome can have a mortality rate as high as 44%. Upside-down stomach accounts for less than 5% of hiatal hernias and can lead to incarceration and volvulus.

**Case Report:**

An 80-year-old woman presented to the emergency department with sudden onset, severe epigastric pain. Physical examination revealed normal vital signs with mild epigastric tenderness. Imaging obtained revealed a large hiatal hernia and findings concerning for esophageal perforation. The patient was started on 3.375 grams of intravenous piperacillin/tazobactam, and transfer to a tertiary care facility was initiated. After transfer, esophagography confirmed a perforation near the gastroesophageal junction and findings consistent with an upside-down stomach. The patient underwent successful repair of the esophageal perforation and gastropexy followed by intensive care unit admission and ultimately discharge.

**Conclusion:**

Boerhaave syndrome and upside-down stomach are two conditions with high associated morbidity and mortality requiring prompt intervention. Information obtained in the history and physical examination including acute onset of chest pain after vomiting, tachypnea, subcutaneous emphysema, and hypoxia can assist in the diagnosis of the described pathologies. These signs and symptoms can be subtle on examination but are important in raising clinical suspicion for an otherwise rare etiology for acute onset chest pain.

## INTRODUCTION

Spontaneous esophageal rupture, or Boerhaave syndrome, is rare with an incidence as low as 3.1 per one million people per year and a mortality rate that triples with a delay in diagnosis of 48 hours from symptom onset.^1^^,^^2^ This condition was first described in a patient who vomited after a large meal and subsequently developed chest pain by Hermann Boerhaave, a Dutch professor of medicine.^1^ Upside-down stomach or a type IV hiatal hernia is the rarest form of hiatal hernia, accounting for less than 5% of all hiatal hernias.^3^

This combination of pathology—Boerhaave syndrome in the setting of an upside-down stomach—has only been described once previously in the surgical literature.^4^ Each condition can individually lead to significant morbidity and mortality. Upside-down stomach has a high documented risk of incarceration, can lead to esophageal outlet obstruction and perforation, and has previously been suggested as a contributing factor to the development of spontaneous esophageal rupture.^4^^,^^5^ Boerhaave syndrome, if not urgently diagnosed and treated, can rapidly lead to mediastinitis and septic shock with a mortality rate of up to 44%.^6^

## CASE REPORT

An 80-year-old woman with a history of gastroesophageal reflux and hypertension presented to the emergency department (ED) with sudden onset, severe, sharp epigastric pain with radiation to her back. The pain began after an episode of emesis immediately following the ingestion of polyethylene glycol approximately 11 hours prior to arrival. Upon her presentation to the ED, the patient was in apparent distress secondary to pain; however, her vital signs were normal with a temperature of 36.7° Celsius (C), blood pressure of 142/65 millimeters of mercury, heart rate of 80 beats per minute, and respiratory rate of 18 breaths per minute. Physical examination revealed only mild epigastric tenderness. No subcutaneous emphysema was present in the tissue overlying the neck or chest. Due to the patient’s acute distress and comorbidities, a broad differential was considered for evaluation of critical etiologies of her clinical presentation including aortic dissection, bowel perforation, esophageal rupture, acute coronary syndrome, mediastinitis, bowel obstruction, and pancreatitis.

**Image 1.**
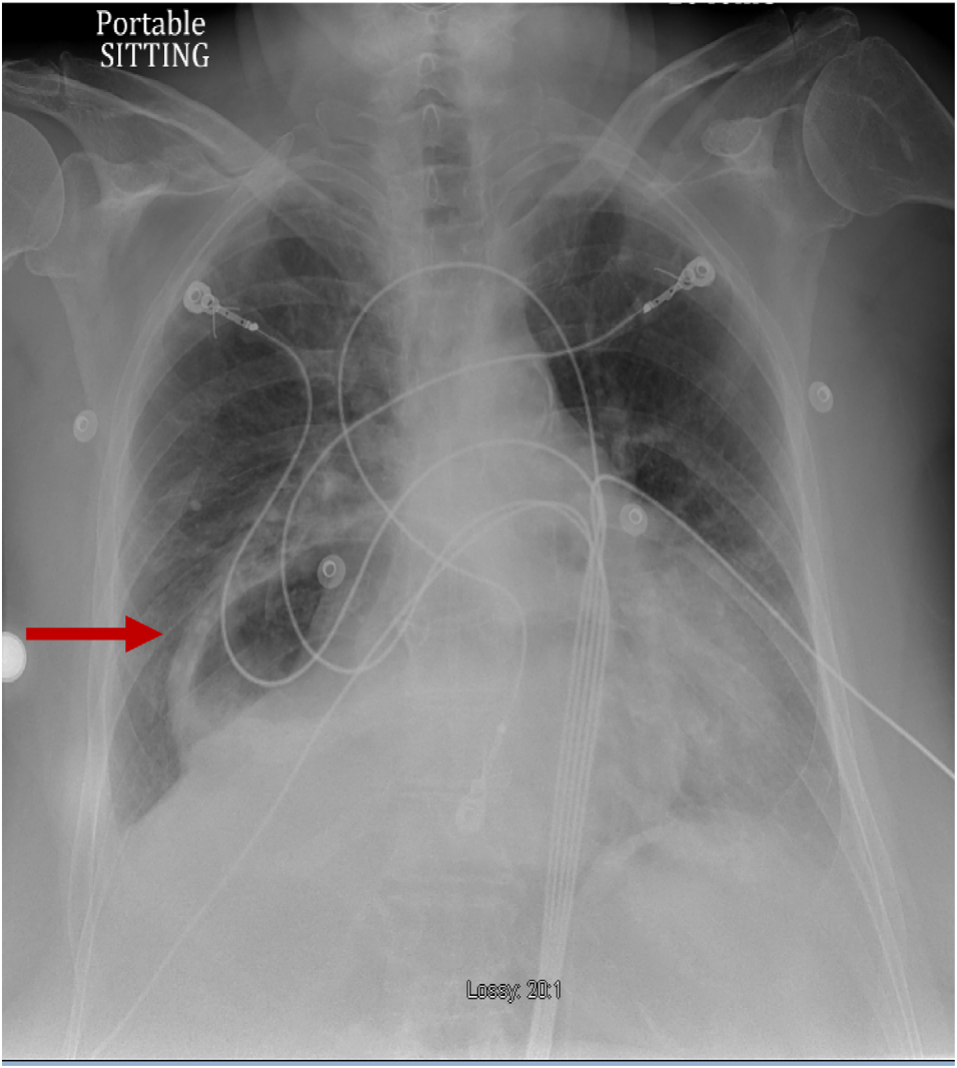
Anterior-posterior chest radiograph demonstrating large hiatal hernia with displacement of the gastric bubble (arrow) from left to right.

**Image 2.**
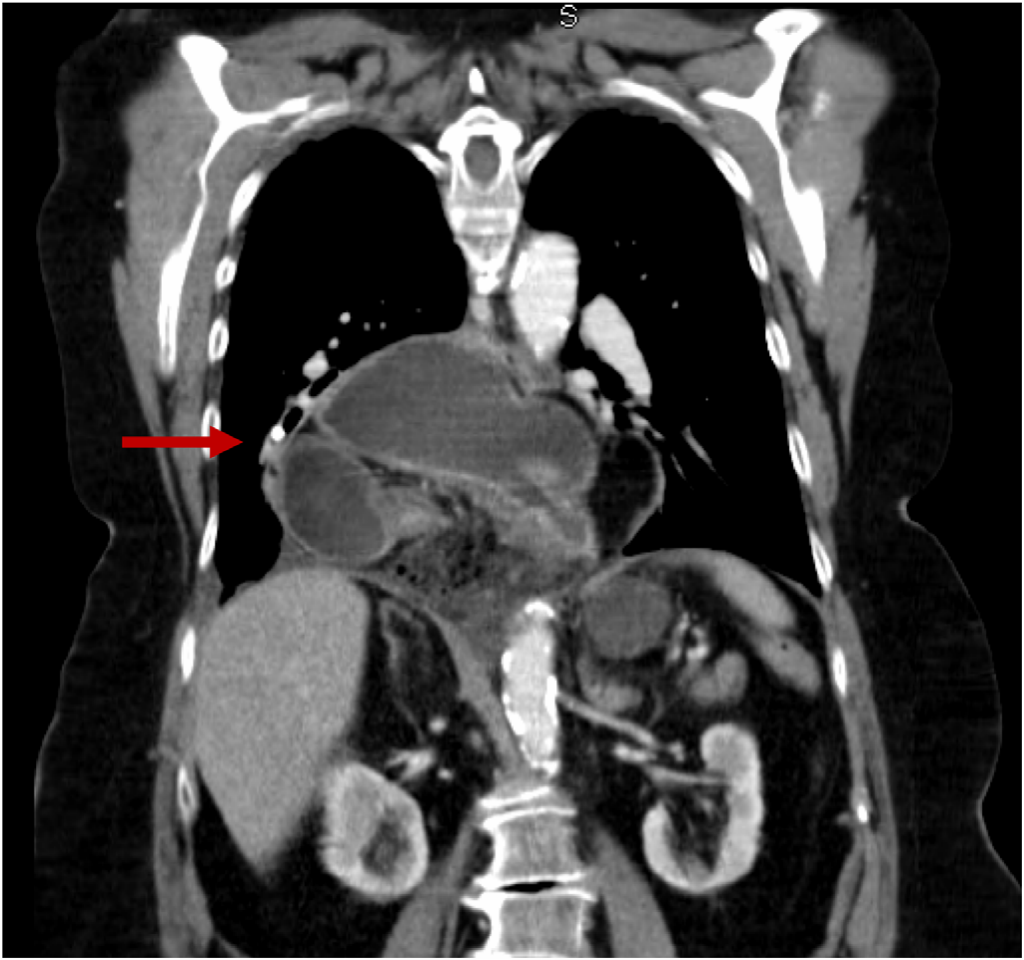
Coronal view of computed tomography of the chest/abdomen/pelvis showing large hiatal hernia (arrow).

**Image 3.**
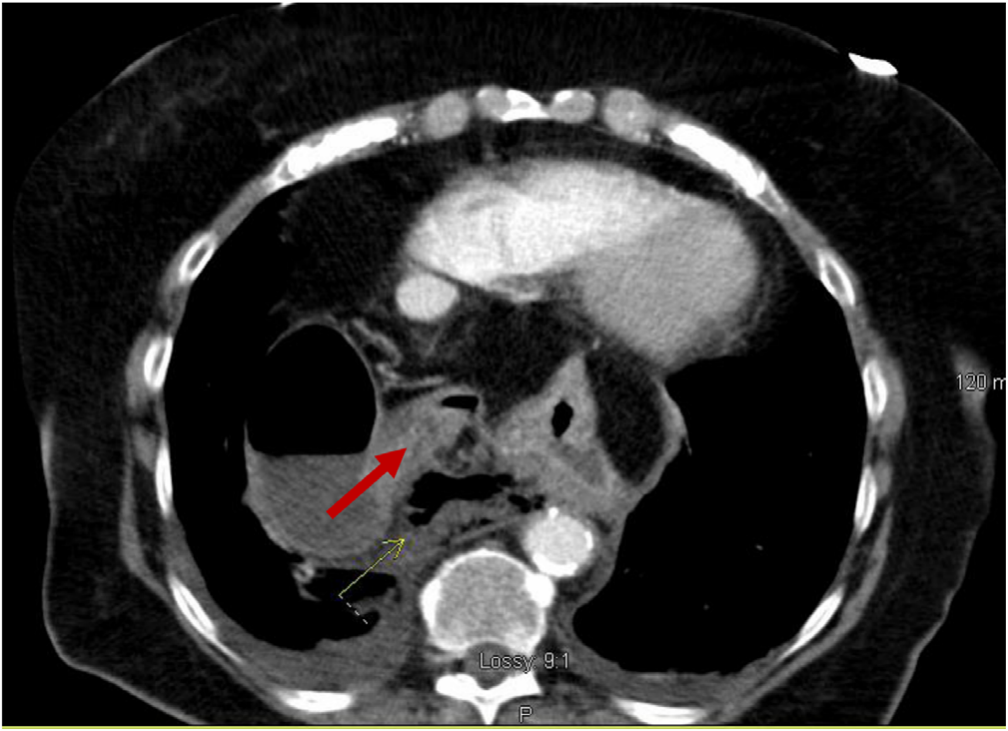
Transverse view of computed tomography of the chest/abdomen/pelvis with an arrow indicating partially fluid-filled collection posterior to a large hiatal hernia suspicious for esophageal perforation.

A chest radiograph (CXR) (Image 1) showed a large hiatal hernia with trace bilateral pleural effusions and displacement of the gastric bubble to the right. Computed tomography (CT) with contrast of the chest/abdomen/pelvis (Images 2 and 3) demonstrated a large hiatal hernia with most of the stomach in the chest, associated volvulus, bilateral pleural effusions, right greater than left, and a complex, partially fluid-filled collection along the posterior aspect of the hiatal hernia just above the diaphragm. Laboratory studies revealed neutrophilic leukocytosis with 83.7 % neutrophils and a white cell count of 16.4 × 10^9^ per liter (L) (4.8–10.8 × 10^9^/L) and elevated lactic acid of 2.3 millimoles per liter (mmol/L) (0.5–2.2 mmol/L). The patient was given 3.375 g of piperacillin/tazobactam intravenously for coverage of gastrointestinal flora, and transfer to a tertiary care facility was initiated. While in the ED, the patient developed room air hypoxia, which resolved with three liters of oxygen therapy via nasal cannula.

After transfer, a contrast esophagram was performed and showed a perforation near the gastroesophageal junction. The patient underwent an exploratory laparotomy, which confirmed the presence of an esophageal rupture near the squamocolumnar junction and revealed the presence of a giant hiatal hernia with an associated upside-down stomach. Surgical repair of the esophageal perforation and gastropexy was performed. The patient was admitted to the surgical intensive care unit for a total of five days during which she had an episode of atrial fibrillation with rapid ventricular response that resolved after metoprolol and diltiazem.

On day five of her hospitalization, she was transferred to a cardiac telemetry unit after being weaned off supplemental oxygen. She was continued on intravenous (IV) fluconazole 400 milligrams (mg) daily, piperacillin/tazobactam 3.375 g every eight hours, and vancomycin dosed and monitored by pharmacy for a total of 14 days. Multiple esophagrams were performed, which revealed a persistent leak from the esophagus; however; this resolved spontaneously with monitoring throughout the remainder of the hospital course. The patient was ultimately discharged on hospital day 18 with continued antibiotic therapy on amoxicillin-potassium clavulanate 600–42.9 mg twice daily for seven days. After discharge, the patient was seen for multiple follow-up visits, with the last follow-up occurring approximately 16 months after her original presentation.

## DISCUSSION

Esophageal rupture is a rare condition with high morbidity and mortality that is iatrogenic in nature in approximately 70% of cases.^2^ The spontaneous form of this condition is estimated to account for between 15–30% of cases.^1^^,^^7^^,^^8^ Boerhaave syndrome is thought to be caused by a sudden increase in intraesophageal pressure leading to a transmural tear through the esophageal tissue.^4^ Although it is important to maintain a comprehensive differential diagnosis, physicians must have a high suspicion for this condition as it is frequently misdiagnosed as perforated ulcers, myocardial infarction, or pulmonary emboli.^4^

This condition is an essential diagnosis for emergency physicians due to a mortality rate reaching as high as 44%. Risk factors for developing spontaneous esophageal rupture include male gender and alcohol abuse.^1^ As described in the case above, there have been documented cases associated with polyethylene glycol ingestion for colonoscopy preparation. This preparation requires ingesting a large amount and can lead to forceful vomiting.^9^ In patients with Boerhaave syndrome, the most common presenting complaint is pain that is usually associated with the site of perforation and can occur in the neck, chest, or abdomen.^2^ This may be associated with vomiting, painful swallowing, and voice change, or fever and physical examination may reveal the presence of tachycardia, tachypnea, and subcutaneous emphysema.^1^^,^^2^

A CXR may show evidence of perforation; however, CT is preferred due to the lower sensitivity of plain radiography. If suspicion remains high for the condition, a contrast esophagram is the preferred diagnostic modality. Barium should be avoided due to the possible development of mediastinitis if an esophageal leak is present; instead, water-soluble contrast should be used.^1^ Although not commonly included in the standard diagnosis of Boerhaave syndrome, bedside point-of-care ultrasound has been used in specific cases. Findings on ultrasound include the presence of free fluid in the upper quadrants of the abdomen and air within the pericardium blocking the normal visualization of cardiac windows.^10^

Although the patient in this case did require surgical intervention, this is not true of all esophageal perforations. For non-operative management, patients must have small defects without significant involvement of structures outside the esophagus. These patients are treated with IV antibiotics for at least 7–10 days and supportive care measures including cardiac monitoring, supplemental oxygen, if necessary, and adequate analgesia. According to a 2010 article by Kaman et al, there are no clear recommendations for patients who should undergo surgical intervention although it likely includes, “early postemetic perforation, hemodynamic instability, intra-abdominal perforation, extravasations of contrast into adjacent body cavities and presence of underlying malignancy, obstruction or stricture in the region of the perforation and surgically fit patient.”^2^

Factors that increase the morbidity and mortality of this condition include time to diagnosis, size of the defect, cause of the defect, and association with neutrophilic leukocytosis.^6^^,^^8^ Possibly the most important factor is the time to diagnosis. A delay in diagnosis leads to an increased possibility of developing mediastinitis and sepsis secondary to the leakage of gastric enzymes and gastrointestinal flora. As little as a 48-hour delay can lead to a three-fold increase in mortality.^2^

Upside-down stomach is a type IV hiatal hernia, most commonly caused by the weakening of the diaphragmatic crura, which accounts for less than 5% of all diagnosed hiatal hernias.^3^^,^^5^ Although some patients may be asymptomatic, approximately one-third will develop life-threatening complications including volvulus, incarceration, perforation, severe gastric bleeding, and gastric ischemia.^3^^,^^4^ Surgical repair, often laparoscopic, is recommended urgently; however, emergent repair is only recommended when complications have occurred, due to the increased risks associated with emergent surgical repairs.^3^^,^^5^ In a previously described case of combined upside-down stomach and esophageal rupture, it was proposed that the large hiatal hernia resulted in blockage of the gastric outlet leading to forceful vomiting and ultimate rupture.^4^

## CONCLUSION

Emergency physicians must consider broad differential diagnoses in all patients to ensure that time-sensitive diagnoses of conditions are made even in those patients who present initially stable. In the above case, the patient presentation of abrupt onset epigastric pain immediately after an episode of emesis following the ingestion of polyethylene glycol led to rapid diagnosis of these rare conditions. Although the patient did decompensate in the ED with the development of tachypnea and hypoxia, rapid management of her condition with antibiotic therapy, oxygen administration, prompt transfer, and surgical intervention led to a favorable outcome.

## References

[1] Turner AR Turner SD . Boerhaave syndrome. In: StatPearls. Stat Pearls Publishing: Treasure Island, FL, 2022. Available at: https://www.ncbi.nlm.nih.gov/books/NBK430808/.

[2] Kaman L Iqbal J Kundil B et al . Management of esophageal perforation in adults. Gastroenterol Res; 2010;3(6):235–44. 10.4021/gr263w PMC5139851 27942303

[3] Schiergens TS Thomas MN Hüttl TP et al . Management of acute upside-down stomach. BMC Surg. 2013;13:55. 24228771 10.1186/1471-2482-13-55 PMC3830558

[4] Saito S Hosoya Y Kurashina K et al . Boerhaave’s syndrome in a patient with an upside down stomach: a case report, Int. J. Surg. Case Rep. 2016;19:51–4. 10.1016/j.ijscr.2015.12.016 PMC4756090 26710329

[5] Merza N Lung J Bazzaz O et al . Rare case of upside-down stomach in advanced hiatal hernia. SRCCC. 2019;7(31):52–5.

[6] Zimmermann M Hoffmann M Jungbluth T et al . Predictors of morbidity and mortality in esophageal perforation: retrospective study of 80 patients. Scand J Surg. 2017;106(2):126–32. 27334795 10.1177/1457496916654097

[7] Søreide JA Konradsson A Sandvik OM et al . Esophageal perforation: clinical patterns and outcomes from a patient cohort of Western Norway. Dig Surg. 2012;29(6):494–502. 23392348 10.1159/000346479

[8] Kim JD . Prognostic factors of esophageal perforation and rupture leading to mortality: a retrospective study. J Cardiothorac Surg. 2021;16(1):291. 34627308 10.1186/s13019-021-01680-y PMC8502388

[9] Yu JY Kim SK Jang EC et al . Boerhaave’s syndrome during bowel preparation with polyethylene glycol in a patient with postpolypectomy bleeding. World J Gastrointest Endosc. 2013;5(5):270–2. 23678383 10.4253/wjge.v5.i5.270 PMC3653029

[10] Derr C Drake JM . Esophageal rupture diagnosed with bedside ultrasound. Am J Emerg Med. 2012;30(9):2093.e1–3. 10.1016/j.ajem.2011.12.036 22386338

